# Transorganogenesis and transdifferentiation in *C. elegans* are dependent on differentiated cell identity

**DOI:** 10.1016/j.ydbio.2016.09.020

**Published:** 2016-10-04

**Authors:** Misty R. Riddle, Erik A. Spickard, Angela Jevince, Ken C.Q. Nguyen, David H. Hall, Pradeep M. Joshi, Joel H. Rothman

**Affiliations:** a Department of Molecular, Cellular, and Developmental Biology, and Neuroscience Research Institute, University of California, Santa Barbara, CA 93106, USA; b Department of Neuroscience, Albert Einstein College of Medicine, Bronx, NY 10461, USA; c School of Biological Sciences, University of Auckland, Auckland 1010, New Zealand

**Keywords:** *C. elegans*, Reprogramming, Transdifferentiation, Transorganogenesis, GATA transcription factor, PHA-4/FoxA transcription factor

## Abstract

The differentiated cell identities and structure of fully formed organs are generally stable after their development. In contrast, we report here that development of the *C. elegans* proximal somatic gonad (hermaphrodite uterus and spermathecae, and male vas deferens) can be redirected into intestine-like organs by brief expression of the ELT-7 GATA transcription factor. This process converts one developing organ into another and can hence be considered “transorganogenesis.” We show that, following pulsed ELT-7 expression, cells of the uterus activate and maintain intestine-specific gene expression and are transformed at the ultrastructural level to form an epithelial tube resembling the normal intestine formed during embryogenesis. Ubiquitous ELT-7 expression activates intestinal markers in many different cell types but only cells in the somatic gonad and pharynx appear to become fully reprogrammed. We found that ectopic expression of other endoderm-promoting transcription factors, but not muscle- or ectoderm-promoting transcription factors, redirects the fate of these organs, suggesting that pharyngeal and somatic gonad cells are specifically competent to adopt intestine identity. Although the intestine, pharynx, and somatic gonad are derived from distant cell lineages, they all express the PHA-4/FoxA transcription factor. While we found that post-embryonic PHA-4 is not necessary for pharynx or uterus reprogramming and PHA-4 is not sufficient in combination with ELT-7 to induce reprogramming in other cells types, knock down of PHA-4 during embryogenesis, which abolishes normal pharynx differentiation, prevents pharyngeal precursors from being reprogrammed into intestine. These results suggest that differentiated cell identity determines susceptibility to transdifferentiation and highlight the importance of cellular context in controlling competency for reprogramming.

## 1. Introduction

A major goal in the study of development is to understand how the history of a cell determines its commitment to a final identity. Differentiated cells can be reprogrammed into induced pluripotent stem cells (iPSCs) or directly converted into other differentiated cell types by forced expression of transcription factors (Tachibana et al., 1996; Takahashi and Yamanaka, 2006). The initial identity of the cell in combination with the set of transcription factors it expresses appears to determine the outcome of reprogramming. To what extent does the differentiated state of a cell predispose, or abrogate, its conversion into another cell type?

Induced pluripotent stem cells (IPSCs) that have been obtained from many cell types more readily differentiate back into the lineage from which they were derived, evidence that the ancestral identity of a cell influences its subsequent redifferentiation (Marchetto et al., 2009; Hu et al., 2010). While much effort has been directed at reprogramming fibroblasts directly into distantly related cells (forced transdifferentiation), such as melanocytes (Tachibana et al., 1996) or neurons (Vierbuchen et al., 2010), the extent to which similar approaches can be applied to reprogram a wider variety of cell types remains unclear. It has been proposed that cells closely related in developmental lineage are more readily interconverted, as their expression states differ by a more limited number of factors (Davis et al., 1987; Davis et al., 1990). Cells that are related by lineage or position may also possess fail-safe mechanisms that maintain specific cell identities, thereby blocking their conversion to other cell fates as long as they remain in the same cellular environment. In addition, some cells from diverse embryonic origins can converge on similar identities, indicating that cell lineage, including germ layer origin, is not an inviolable determinant of final identity.

Owing to the fully described, invariant cell lineage and cellular anatomy (Sulston and Horvitz, 1977; Deppe et al., 1978; Sulston et al., 1983b), *Caenorhabditis elegans* is well-suited for investigating how cellular context influences cell reprogramming. In addition to a well-described natural transdifferentiation event that occurs during post-embryonic development, the conversion of a rectal epithelial cell Y to a cholinergic motor neuron PDA (Jarriault et al., 2008), cells can be driven in vivo to change identity by forced ectopic expression of particular transcription factors ( Kalb et al., 1998b; Zhu et al., 1998; Gilleard and McGhee, 2001; Fukushige and Krause, 2005; Yuzyuk et al., 2009). *C. elegans* germline stem cells can also be reprogrammed into differentiated somatic cells by such an approach; however, this transdifferentiation process requires both expression of differentiation-promoting transcription factors and removal of other components, including translational regulators or chromatin remodeling factors (Ciosk et al., 2006; Tursun et al., 2011; Patel et al., 2012).

The somatic cells of the early *C. elegans* embryo are competent to be reprogrammed into cells of any of the three germ layer types by forced expression of single transcription factors (Horner et al., 1998; Zhu et al., 1998; Gilleard and McGhee, 2001; Quintin et al., 2001; Fukushige and Krause, 2005). The period of competency for reprogramming continues through to the end of gastrulation, after which cells commit to particular pathways of differentiation and become refractory to reprogramming. The timing of this “multipotentiality-to-commitment transition” (MCT), which normally occurs during mid-embryogenesis, can be extended somewhat by the removal of chromatin remodeling factors or Notch pathway components (Yuzyuk et al., 2009; Djabrayan et al., 2012); however, major regulators of germ layer identity are generally unable to reprogram cell identity after the MCT. We previously reported that brief expression of ELT-7, a GATA-type transcription factor that controls intestine differentiation (Sommermann et al., 2010), can promote transdifferentiation of cells of the neuromuscular pharynx into intestine-like cells at any time during development or in adulthood (Riddle et al., 2013). While this process results in cells with striking similarity to intestinal cells, they do not assemble into a gut-like organ and hence this process does not reflect transorganogenesis – the conversion of one developing organ type into another.

We report here that organs of *C. elegans* somatic gonads – both the hermaphrodite uterus and spermatheca, and the male vas deferens – can be reprogrammed into intestine following a brief pulse of ectopic ELT-7 expression as late as the terminal stages of gonadal organogenesis. The ultrastructure of the redirected uterus is virtually indistinguishable from that of the normal intestine and animals with a transfated uterus appear to contain two parallel intestinal organs. Earlier reports indicated that other GATA transcription factors in the endoderm regulatory cascade, END-3 and ELT-2, cannot reprogram cells after the MCT stage during mid-embryogenesis (unpublished observations, Fukushige et al., 1998; Djabrayan et al., 2012). In contrast, we found that END-3 and ELT-2 can, in fact, induce transdifferentiation post-embryonically, revealing that ELT-7 is not uniquely capable of cellular reprogramming. While the pharynx, somatic gonad, and intestine are distantly related in cell lineage, all three express the PHA-4/FoxA transcription factor (Kalb et al., 1998a). FoxA and GATA transcription factors collaborate to control digestive-tract development across metazoa and function as “genetic potentiators” to specify endodermal cell identities in mice (Reuter, 1994; Zaret, 1999; Lengyel and Iwaki, 2002; Zorn and Wells, 2009). We found that post-embryonic PHA-4 expression is neither necessary in the pharynx or uterus, nor sufficient outside of these organs, for transdifferentiation by ELT-7. However, normal pharynx differentiation orchestrated by PHA-4 in the embryo is required for the later transdifferentiation of the pharynx into intestine. Thus, our findings reveal that organogenesis can be redirected in vivo by a single transcription factor and that the prior cellular history and differentiation state are important for determining susceptibility to transdifferentiation.

## 2. Results and discussion

### 2.1. Multiple GATA transcription factors in the endoderm regulatory cascade can reprogram differentiated pharynx cells into intestine-like cells

**Fig. 1:**
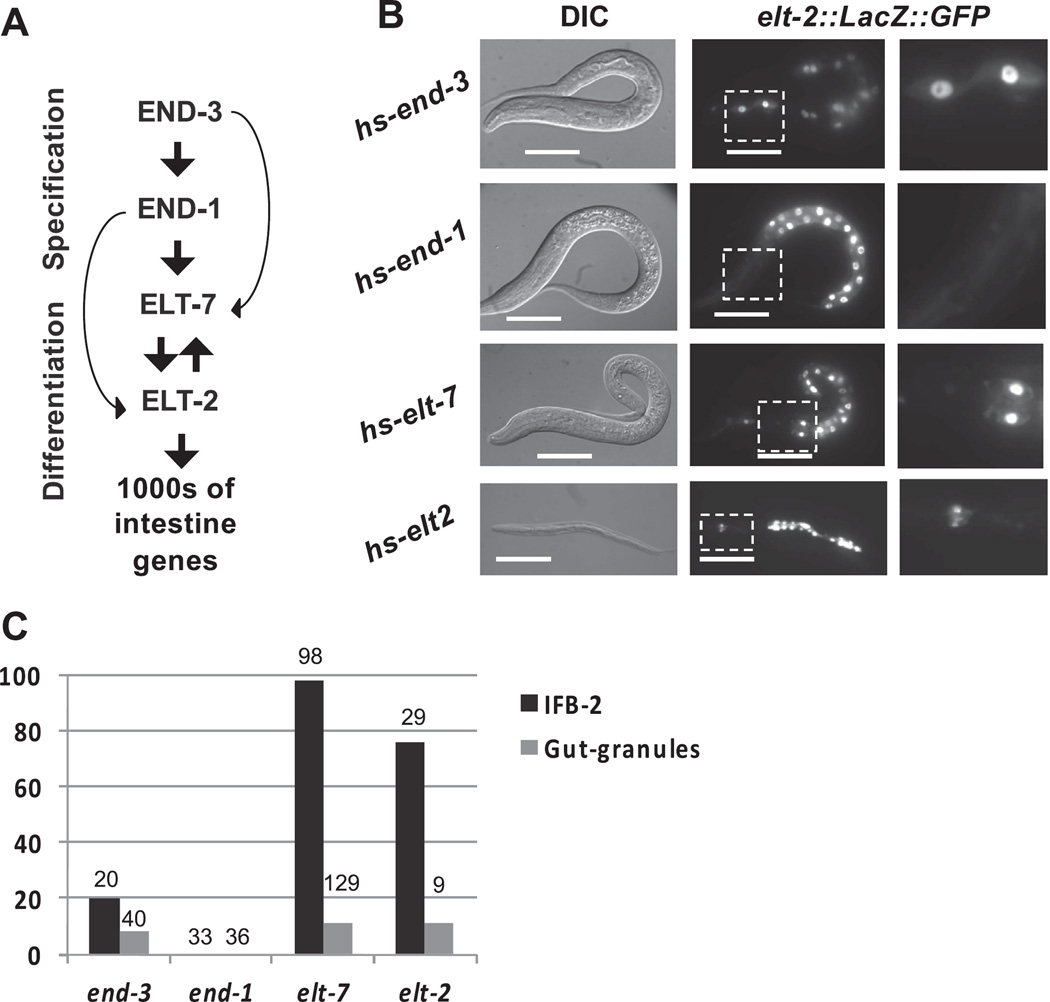
Ectopic expression of multiple endoderm-promoting GATA transcription factors induces pharynx-to-intestine transdifferentiation. (A) GATA transcription factors that control the development of the *C. elegans* intestine. END-3 and END-1 are transiently expressed in the early embryo, specify the endoderm progenitors, and activate expression of ELT-7 and ELT-2. ELT-7/2 in turn activate and maintain robust expression of the genes required for intestine formation and function through cross and auto-activation. (B) Worms after brief heat-shock-driven ubiquitous expression of END-3, END-1, ELT-7, or ELT-2. Activation of intestine differentiation in the pharynx is evidenced by maintained *elt-2* reporter expression anterior to the normal intestine(anterior is left, last panel shows boxed region in middle panel, DIC differential interference contrast, scale bars 20 µm). (C) Percentage of worms with immunoreactive IFB-2 intestine-specific intermediate filament in the pharynx, and gut-specific granules in the pharynx after ectopic GATA factor expression (total number of worms is indicated above each bar).

Cells of the *C. elegans* pharynx and intestine are distinct in cell lineage, morphology, function, and gene expression (Horner et al., 1998; Maduro and Rothman, 2002; Mango, 2007; Sommermann et al., 2010). During early embryogenesis, a rapidly deployed cascade of GATA-type transcription factors specifies the endoderm (END-1 and END-3) and subsequently activates intestinal differentiation (ELT-2 and ELT-7; Fig. 1A). Positive cross-regulation between ELT-2 and ELT-7 appears to maintain expression of all intestine-expressed genes throughout development and adulthood (Maduro and Rothman, 2002; Maduro et al., 2005; Sommermann et al., 2010; Wiesenfahrt et al., 2016). We previously found that brief expression of ELT-7 via a ubiquitously activated heat-shock promoter reprograms differentiated post-mitotic pharyngeal cells into intestine-like cells at any stage of development (Riddle et al., 2013). As reported previously, we confirmed that END-1 is not capable of activating expression of late markers of intestine differentiation in the pharynx (Fig. 1B; Zhu et al., 1997; Zhu et al., 1998; Riddle et al., 2013). As ELT-7 is the smallest of the endoderm GATA factors, it was conceivable that either its size, or some unique structural characteristic, endows it with the ability to access promoters contained in otherwise inaccessible chromatin within differentiated cells. Indeed, earlier studies showed that neither the endoderm-specification factor END-3, nor the gut differentiation factor ELT-2, were able to activate widespread ectopic endoderm development in embryos after the MCT (Fukushige et al., 1998; Djabrayan et al., 2012). However, we found that, like ELT-7, both END-3 and ELT-2 are, in fact, capable of reprogramming differentiated pharynx cells, albeit somewhat less efficiently than ELT-7 (Fig. 1B–C). A 15-min pulse of either END-3 or ELT-2 expression induces stable endogenous ELT-2 expression (Fig. 1B), as well as dramatic alteration of the entire cellular architecture as indicated by expression and apical localization (not shown) of immunoreactive intestine-specific intermediate filament IFB-2, altered nuclear morphology, and formation of “gut granules” which are a morphological hallmark of late intestinal differentiation (Fig. 1C). Thus, ELT-7 is not uniquely capable of reprogramming differentiated cells and in contrast to earlier reports (Fukushige et al., 1998; Djabrayan et al., 2012) the capacity of transcription factors to override the MCT and reprogram even fully differentiated post-mitotic cells is distributed among most of the endoderm regulatory factors.

### 2.2. Reprogramming of the somatic gonad into intestine

**Fig. 2:**
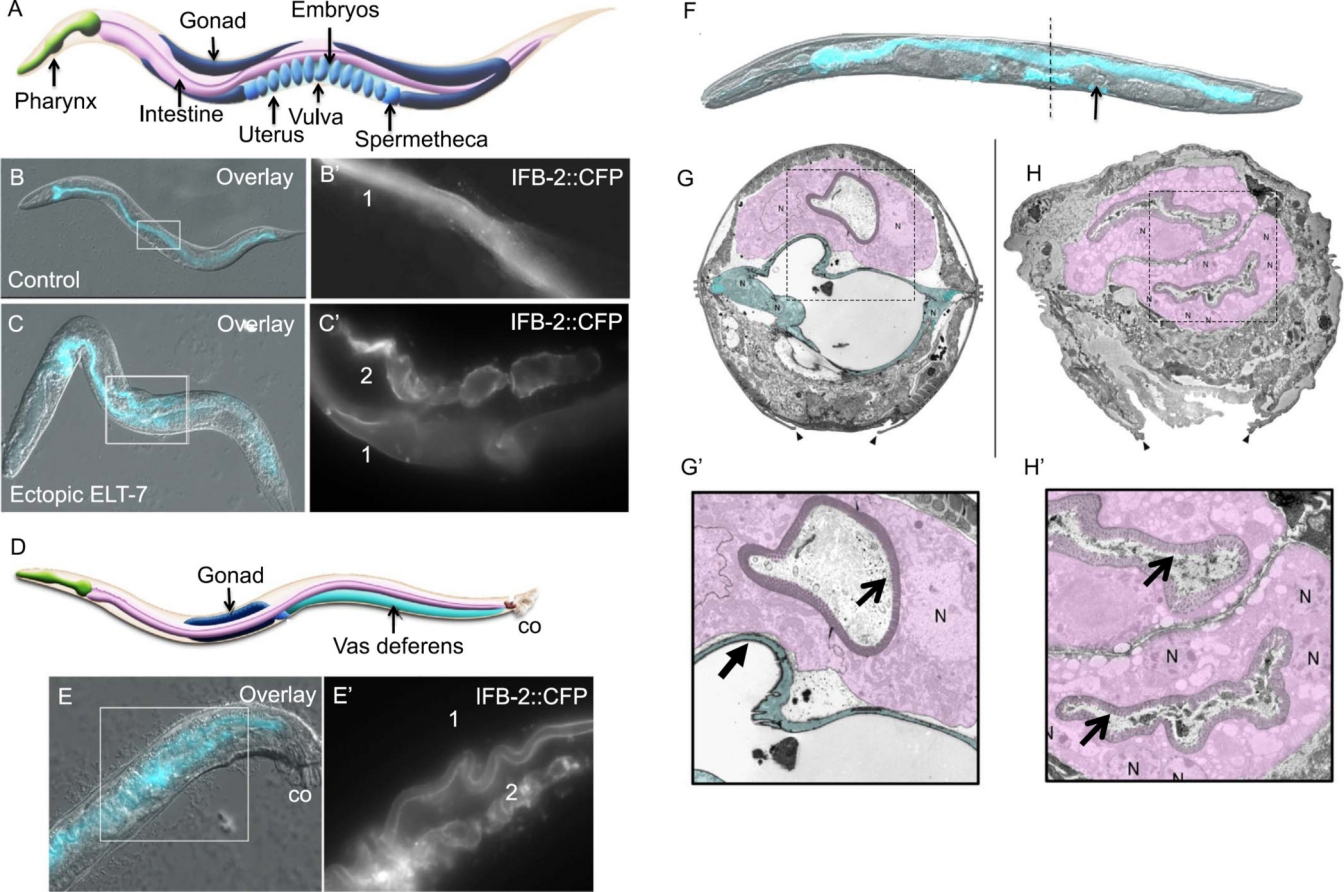
Transorganogenesis of the somatic gonad into intestine. (A) Diagram of adult *C. elegans* hermaphrodite (approximately 1 mm in length) showing the neuromuscular pharynx, intestine, and the two “U” shaped gonad arms that are connected by two spermathecae and a uterus. (B, B’) Expression and localization of intestine-specific intermediate filament protein (IFB-2::CFP) that lines the single intestinal lumen “1” in an adult hermaphrodite. (C, C’) IFB-2::CFP expression 48 h after a 15-min pulse of ELT-7 expression at the L4 stage. Additional IFB-2-lined lumen “2” is seen in the uterus. (D) Diagram of adult *C. elegans* male (approximately 0.8 mm in length) showing the vas deferens (co; copulatory organ). (E, E”) IFB-2::CFP in a male 48 h after brief ectopic ELT-7 expression. IFB-2 expression is visible in the intestine “1” and vas deferens “2”. (F) Hermaphrodite 48 h after ectopic ELT-7 induction at the L4 stage (DIC and CFP overlay). An embryo is retained in one spermatheca (arrow). Dotted line indicates approximate transverse section of micrograph shown in H. (G) Transmission electron micrograph of a transverse section at the vulva of an L4 stage hermaphrodite (intestine is shaded purple and uterus is shaded blue, arrowheads point to the edges of the vulval opening, N, cell nucleus). (H) Transmission electron micrograph of a transverse section at the vulva of an L4 stage hermaphrodite 48 h after ectopic ELT-7 expression; the two intestine-like epithelial tubes are shaded in purple. The normal intestine (upper epithelial tube) shows 1–2 nuclei per cross-section, but the converted uterine tissue, which is derived from many more cells, shows many nuclei. (G’, H’) Magnified region of micrographs in G and H showing the smooth lumen of the uterus (closed arrow) and rough intestinal lumen that is lined with microvilli (open arrows). Cartoons are reprinted with permission from Altun and Hall (2009) (www.wormatlas.org).

To investigate the dynamics of pharynx remodeling following ectopic ELT-7 expression at each *C. elegans* larval stage (L1-L4), we analyzed expression of CFP-tagged intermediate filament protein, IFB-2 (Husken et al., 2008). IFB-2 is a component of the terminal web structure that localizes specifically to the apical surface of intestinal cells and is never detected outside of the intestine (Fig. 2B; Fukushige and McGhee, 2001; Maduro and Rothman, 2002; Husken et al., 2008). We observed IFB-2::CFP on the apical surface of pharyngeal cells within 24 h after brief ectopic ELT-7 expression at any developmental stage or adulthood, consistent with our previous observations of immunoreactive IFB-2 (Figs. 1C, 8D; Riddle et al., 2013). Unexpectedly, we found that when ELT-7 expression is activated at late postembryonic stages, IFB-2 is expressed in the mid-body of the worm, more broadly distributed than previously detected (as we had focused earlier on the head region of decapitated adults to allow for antibody penetration). Examination of the intact animal revealed abundant IFB-2::CFP that localized to an intestine-like lumen in the region of the gonad 48 h after a 15-min pulse of ectopic ELT-7 expression at any time between the L3 and mid-L4 stage (Fig. 2C). The IFB-2-lined lumen is parallel and ventral to the intestine and subjacent to the vulva, consistent with the normal position of the proximal gonad (Fig. 2A – C).

The fully developed gonad comprises two reflexed arms containing differentiating germ cells encased in somatic sheaths. The arms terminate in spermathecae that connect to the vulva via the uterus (Fig. 2A). The gonad develops post-embryonically; the uterine cell lineage is completed at the early L4 stage and uterine morphogenesis continues to the late L4 stage (Kimble and Hirsh, 1979; Newman et al., 1996). The cell types of the uterus do not exhibit characteristics similar to intestine at any developmental stage (Newman et al., 1996; Hall et al., 1999). We found that the ectopic gut-like organ arising after pulsed ELT-7 expression connects the two gonad arms via the spermathecae in the position of the normal uterus (Fig. 2A–C). Fertilized oocytes normally travel from the spermathecae into the uterus and developing embryos are laid when they contain approximately 28 cells. We found that 48 h after a 15-min pulse of ELT-7 expression between the L3 and adult stages, the function of the uterus is abolished; the few embryos that are sometimes produced after ectopic ELT-7 expression are retained in the spermatheca or the proximal gonad, apparently unable to enter the uterus (Fig. 2F, arrow).

**Fig. 3:**
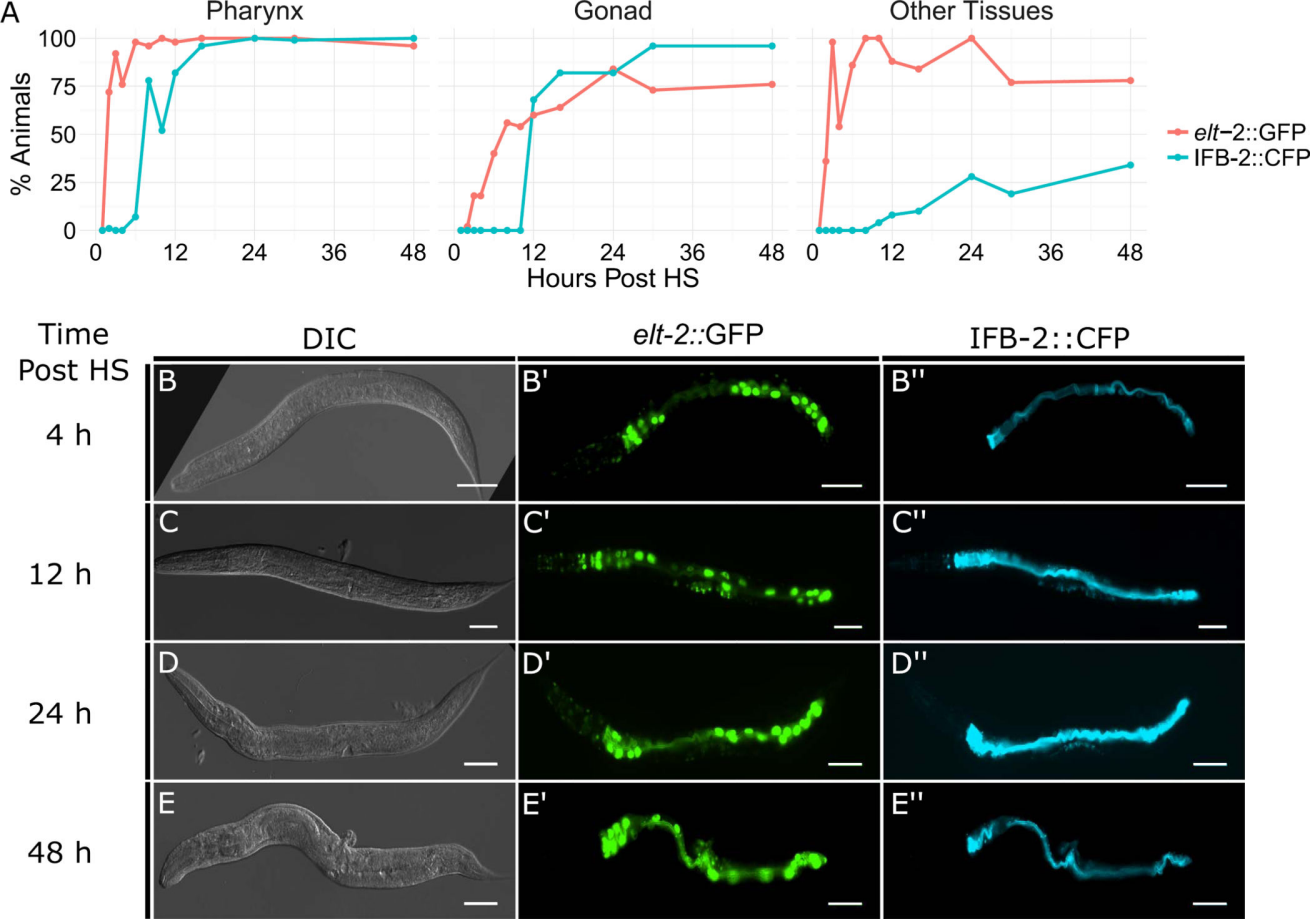
Time line of gut-specific marker expression following activation of ubiquitous *elt-7* expression at early L4 stage. (A) Percentage of worms expressing *elt-2::GFP* and IFB-2::CFP in the pharynx (left), somatic gonad (center), and other tissues and organs excluding intestine (right). (B–E”) Representative DIC and epifluorescence images of worms at progressive intervals following activation of ELT-7 expression (scale bar, 50µM).

**Fig. 4:**
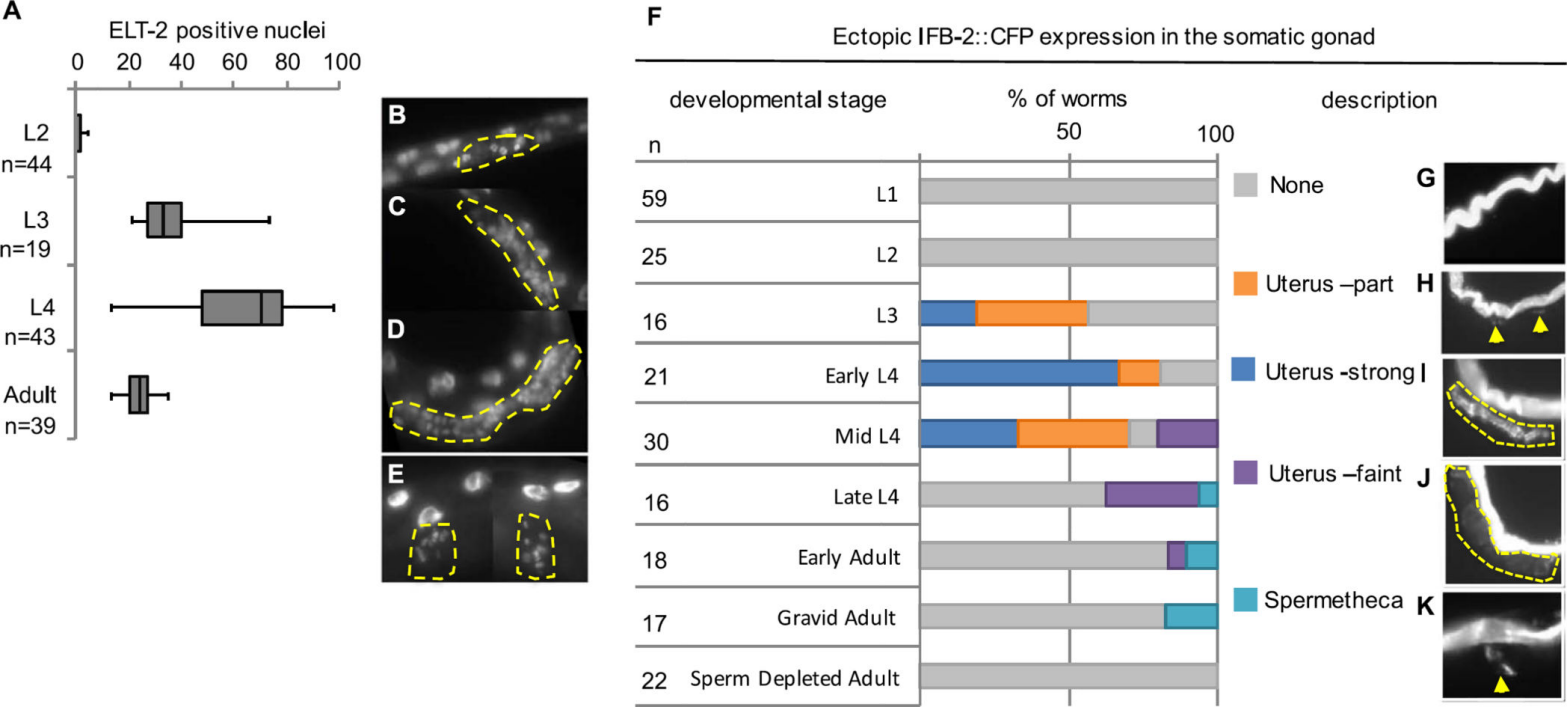
Organogenesis of the somatic gonad can be redirected into intestine at both proliferative and post-mitotic stages. (A) Comparison of the number of immunoreactive ELT-2 nuclei in the somatic gonad after ectopic ELT-7 expression at the indicated stage. n, number of worms. ELT-2-expressing nuclei in the proximal gonad (yellow outline) 48 h after pulsed ELT-7 expression at the L2 (B), L3 (C), and L4 (D) stage. (E) ELT-2-expressing nuclei in the spermatheca (yellow outline) after ectopic ELT-7 expression at the adult stage. (F) Percentage of worms with IFB-2::CFP expression in the somatic gonad 48 h after pulsed ELT-7 expression at the indicated stages determined by worm length and vulval morphology. n, number of worms. (G–H) Typical example of the described phenotypes with yellow arrows and dotted lines demarcating the region of ectopic IFB-2::CFP expression. (G, none) no ectopic IFB-2. (H, Uterus-part) Some ectopic IFB-2 that does not form a complete lumen. (I, Uterus-strong) IFB-2 expression similar to intestine that outlines an intestine-like lumen. (J, Uterus-faint) Faint IFB-2 expression that outlines a wider more uterus-like lumen; (K, Spermathecae) ectopic IFB-2 in one or both spermathecae.

The similarity in architecture of the normal gut and the gut-like organ present in the region normally occupied by the uterus, and particularly the luminal structure, was evident from 3-D reconstruction (e.g., see Supplemental Movie 1). The intestinal morphology arising in the somatic gonad is particularly striking at the ultrastructural level observed by electron microscopy (Fig. 2G–H); in the transformed organ, the normally smooth and flat morphology of the uterus has morphed into an intestine-like endotube with a characteristic terminal web structure and highly organized microvilli that are indistinguishable from those of the normal intestine (Fig. 2G’–H’). The nuclear morphology also resembles that of the normal intestine; however, there are a greater number of nuclei, corresponding to the number of nuclei present in the uterus in late-stage animals. Consistent with these dramatic morphological changes, cells of the uterus maintain expression of endogenous ELT-2, long after the brief pulse of ELT-7, suggesting a stably activated transcriptional program that drives robust intestine-specific expression (Fig. 3A–E, Fig. 4A–E). We observed that reprogramming and redirection of organ development could be induced after the cell divisions of the uterus are complete at the early L4 stage, and we refer to this event as “transorganogenesis,” i.e., redirection of the development of one organ, the proximal somatic gonad, into that of another, the intestine. We found that transorganogenesis is not limited to the hermaphrodite proximal somatic gonad: the male vas deferens, which develops from the same cell lineage as the hermaphrodite somatic gonad (Kimble and Hirsh, 1979), similarly undergoes striking transformation into an intestine-like organ in response to ELT-7 expression (Fig. 2D,E).

We examined the onset of ectopic marker expression after ectopic ELT-7 expression from the L3 to mid-L4 stages (Fig. 3). We observed widespread ELT-2::GFP expression in most tissues as early as 4 h, as previously reported (Fig. 3; Riddle et al., 2013). IFB-2 expression is first observed in the pharynx 8–12 h after ectopic ELT-7 expression, and in the somatic gonad at approximately 12 h (Fig. 3A, C). By 48 h, over 95% of worms showed stable ectopic IFB-2 expression in the somatic gonad and pharynx (Fig. 3A, E). The order and timing of intestine gene expression in the transformed cells parallels the events occurring during embryonic development, suggesting redeployment of the embryonic program for gut development. We also observed faint ELT-2 and IFB-2 reporter signal in other tissues in some animals, although this expression was less readily detected (Fig. 3A – E). In subsequent experiments, we chose to assess reprogramming at 48 h after ectopic ELT-7 expression, as this is the time point at which we observed the most striking transformation in cellular phenotype.

We tested whether the proximal somatic gonad can activate intestine development when ELT-7 is expressed at different stages of organogenesis (Fig. 4). The gonad develops post-embryonically from two cells present at hatching that proliferate and differentiate through the four larval stages (L1-L4). While we observed activation of ELT-2 in the somatic gonad precursors in response to ELT-7 expression at the L2 stage (Fig. 4A,B), ectopic gut development did not appear to progress further based on the absence of IFB-2 expression (Fig. 4F). We found that susceptibility of the somatic gonad to transorganogenesis is greatest during the proliferation of the uterine cell lineage, between the L3 and mid-L4 stage (Fig. 4A, C, D, F). During this time, the somatic gonad expands from 25 dividing cells to a total of 142 post-mitotic cells. While ELT-2 was never detected in the gonad under control conditions (n=135), we observed as many as 98 ELT-2-positive nuclei in the somatic gonad following forced ELT-7 expression during the L4 stage (Fig. 4A, D), suggesting that much of the somatic gonad undergoes transdifferentiation into intestine. The ectopic ELT-2-expressing nuclei appear smaller than the endogenous intestinal nuclei at the L4 stage (Fig. 4D). During larval gut development, the gut nuclei undergo several rounds of endoreduplication, which causes them to increase in size. The reprogrammed cells in the somatic gonad appear not to have undergone endoreduplication based on their smaller size, as further supported by analysis of DNA content, which did not indicate an elevated ploidy in these cells (not shown).

We found that IFB-2 was most strongly expressed in the proximal somatic gonad, and delimited a luminal structure that was most similar to that in the *bona fide* gut, when ELT-7 was ectopically expressed between the L3 and mid-L4 stages (Fig. 4I). At the L4 stage, a portion of proximal somatic gonad cells express the EGL-13/SOX domain transcription factor, which is important for late stages of uterine cell differentiation (Hanna-Rose and Han, 1999; Newman et al., 1995). We observed overlap of immunoreactive ELT-2 and EGL-13/SOX reporter following ELT-7 expression at the early L4 stage, providing evidence that differentiating uterine cells activate intestine-specific gene expression (Fig. S1, n=21). Overall, our results suggest that the uterine lineages are most susceptible to reprogramming during the proliferative phase of development (Fig. 4F, I), but that reprogramming can also be induced past early L4 in post-mitotic cells (Fig. 4F).

We found that, while susceptibility of the somatic gonad to transorganogenesis sharply declines following the completion of the uterine cell lineage, evidence of transdifferentiation is not abolished. Pulsed ELT-7 expression during the late-L4 or early adult stage does not result in formation of a second intestine-like lumen, but does result in faint IFB-2 expression that surrounds a wider lumen (Fig. 4J), as well as pronounced IFB-2 and ELT-2 expression in the spermathecae immediately adjacent to the uterus (Fig. 4E, K). The mechanisms that restrict the fate of differentiated uterine cells at the late L4 and adult stage may be relaxed in the adjacent spermathecae. We considered the possibility that the presence of embryos in the uterus may suppress susceptibility to reprogramming; however, we found that the uterus of sperm-depleted hermaphrodites containing no eggs is similarly refractory to transorganogenesis (Fig. 4F, n=22).

We found that although the uterus can be reprogrammed into intestine after uterine cells have become post-mitotic, morphogenesis is nearly complete, and embryos start to form, it appears that once the uterus begins to accumulate embryos, it loses competency for reprogramming. This contrasts with cells of the pharynx, which are competent to be reprogrammed at any stage of development through adulthood (Riddle et al., 2013). As in the hermaphrodite, the male gonad appears to be most susceptible to transorganogenesis at the L3 stage (Fig. 2E) and the adult male gonad is refractory to reprogramming (not shown). The vas deferens develops from the same progenitor cells as the uterus and relies on similar genetic programs (Newman et al., 1996). We hypothesize therefore that the general program for somatic gonad identity may establish a permissive state for reprogramming into intestine.

### 2.3. The pharynx and somatic gonad are specifically competent to transdifferentiate into intestine

We have shown that transdifferentiation, provoked by brief ubiquitous expression of the endoderm-promoting GATA transcription factors, appears to be specific to the pharynx and somatic gonad (Figs. 1 and 2). However, after ubiquitous heat shock promoter-driven expression of END-3, ELT-7, or ELT-2, most cells in the worm transiently express intestinal genes (as indicated by brief *elt-2*::GFP expression, (Riddle et al., 2013)) revealing that intestine-specific GATA factors can function in non-intestinal cell types to activate transcription of their target genes. However, even after prolonged heat shock (up to 1 h, or several 15-min heat shocks spaced by 15–30 min intervals) only the pharynx and somatic gonad cells maintain stable intestine gene expression and undergo transformation into cells with a gut-like morphology. While it is possible that heat stress might play a role in permitting reprogramming, it is clearly not sufficient since all cells experience heat stress and activate *elt-2* transiently, yet only the pharynx and somatic gonad are reprogrammed. What is the context that permits reprogramming in these organs?

**Fig. 5:**
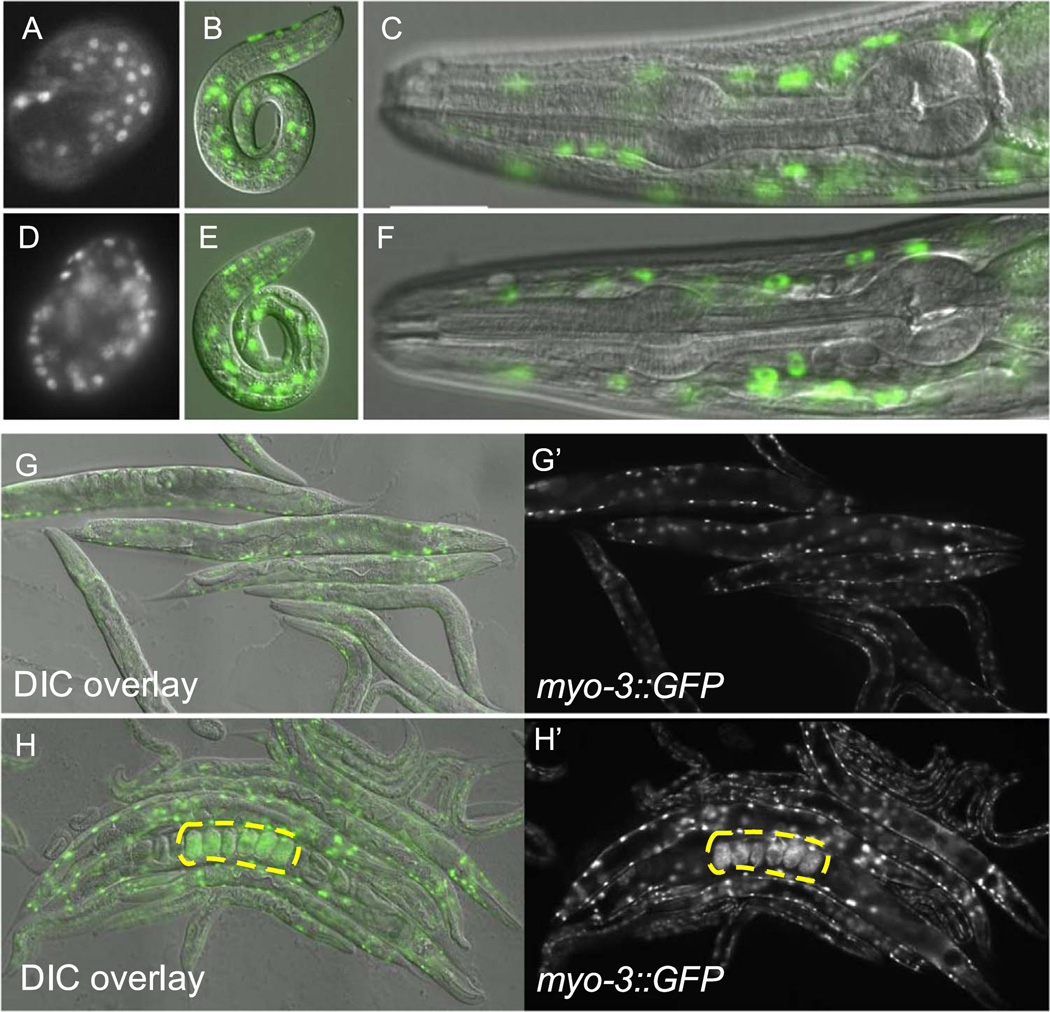
Muscle- and epidermis-promoting transcription factors do not reprogram post-embryonic differentiated cells. (A–C) Expression of *dpy-7::GFP* reporter in epidermal cell nuclei in a *C. elegans* embryo (A, embryos are approximately 50 µM in length), L1 stage worm (B, approximately 250 µM in length DIC with GFP overlay), and head of adult worm (C, approximately 0.1 mm, DIC with GFP overlay). (D) Expression of *dpy-7::GFP* in a representative embryo after forced ubiquitous expression of the epidermal promoting GATA transcription factor ELT-1 (n=6). Expression of *dpy-7::GFP* 48 h after ectopic ELT-1 expression at the L1 (E) and adult (F) stage (n=24). (G-G′) Expression of *myo-3::GFP* reporter in the body wall muscle cells in a group of adult worms. (H–H′) Expression of *myo-3::GFP* reporter in a group of adult worms following ectopic HLH-1 expression. Widespread expression of *myo-3::GFP* is visible in retained embryos (yellow outline), but not adult worms.

The cells of the pharynx and somatic gonad are not broadly developmentally plastic per se. We found that while forced widespread expression of muscle- (HLH-1; Fukushige and Krause, 2005), or epidermis- (ELT-1; Gilleard and McGhee, 2001) promoting transcription factors can reprogram early embryonic cells (Fig. 5D, H), neither is capable of reprogramming differentiated pharynx or somatic gonad cells at post-embryonic stages (Fig. 5 E, F, H). Rather, it appears that the pharynx and somatic gonad are specifically poised to transdifferentiate into intestine. The capacity of these two organs to undergo transdifferentiation does not appear to reflect any lineal relatedness, as the intestine, many of the pharyngeal cells, and the cells forming the somatic gonad are widely separated in cell lineage during the first few embryonic cell divisions (Sulston et al., 1983a).

### 2.4. Normal pharynx differentiation, controlled by PHA-4/FoxA, is required for transdifferentiation

**Fig. 6:**
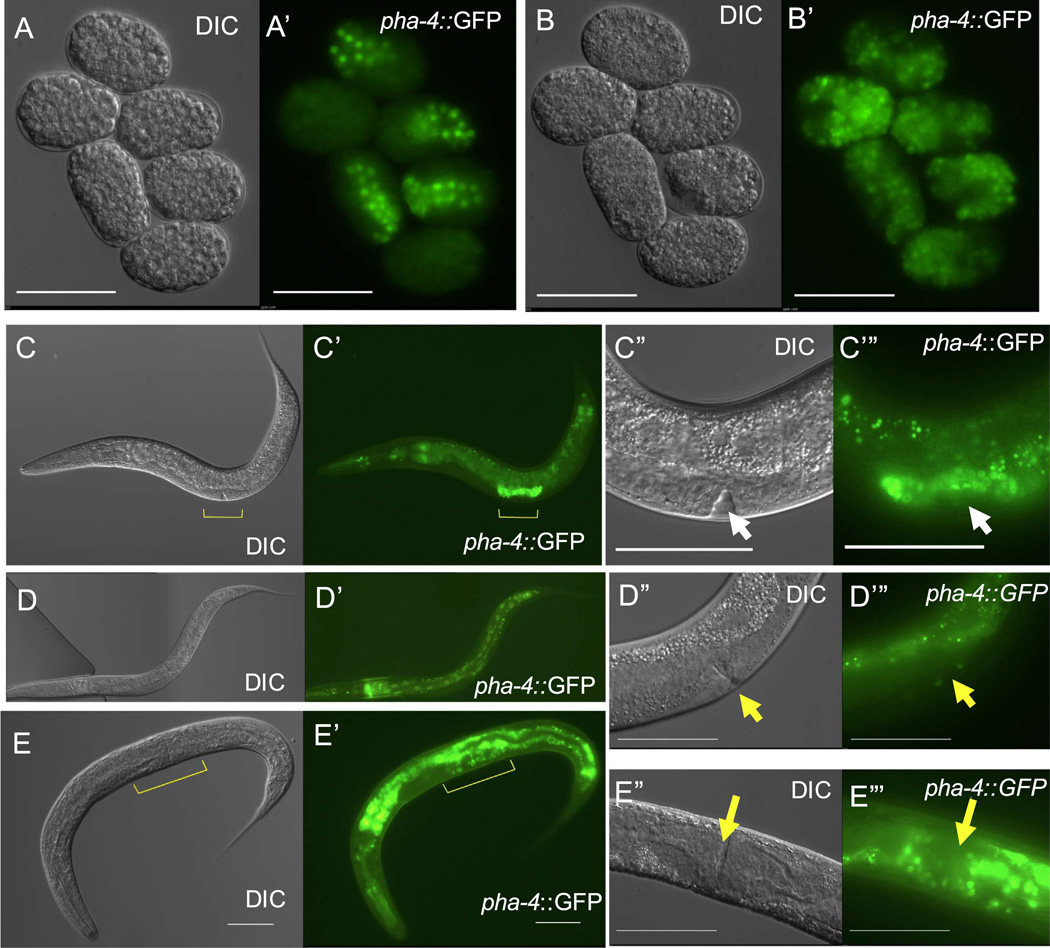
Expression of PHA-4/FoxA transcription factor is maintained in ectopic intestinal cells. (A-B’) *pha-4* reporter expression in six embryos before (A, A’) and 20 h after ectopic ELT-7 expression (B, B’). (C–C’) *pha-4::GFP* expression in L4 stage worm (approximately 600 µM in length). Expression is visible in the pharynx, intestine, and developing proximal gonad (yellow marker). (C”–C”) Magnification of C showing strong *pha-4* expression in the proximal gonad (white arrow, developing vulva). (D–D”’) *pha-4::GFP* expression in an adult worm. (D”–D’”) Magnification of D showing absence of *pha-4* reporter expression in the proximal gonad (yellow arrow, adult vulva). (E–E’”) *pha-4* reporter expression in an adult worm 48 h after ectopic ELT-7 expression at the L4 stage (yellow marker is proximal gonad). (E’-E’”) Magnification of E showing maintained *pha-4*::GFP expression in the proximal gonad of a worm with a fully developed vulva (yellow arrow, n=49, scale bar; 25 µm).

We hypothesized that the pharynx and somatic gonad are susceptible to conversion into intestine because they express a common factor or set of factors that provide the cellular context for intestinal transdifferentiation. A strong candidate is the PHA-4/FoxA transcription factor. PHA-4 is expressed at low levels in the intestine and at high levels in the pharynx beginning in the embryo and continuing through larval development and adulthood (Kalb et al., 1998b). PHA-4 is also transiently expressed in the developing somatic gonad during the L3 and L4 stages (Fig. 6C); after the L4 stage, expression declines sharply and is undetectable in adults (Fig. 6D). These observations prompted us to examine the role of PHA-4 in transdifferentiation.

First, we found that PHA-4 is expressed not only in the cells that are poised to transdifferentiate, but also in those that are converted to intestine-like cells. Ectopic activation of ELT-7 in embryos, before the MCT, induces widespread, stable expression of a *pha-4* reporter throughout the embryo, consistent with expression of *pha-4* in the normal intestine (Fig. 6A and B). Further, we found that after reprogramming, *pha-4* expression is maintained in somatic gonad cells that normally only transiently express *pha-4* (Fig. 6E, n=49).

We reasoned that PHA-4/FoxA, in combination with ELT-7, may be sufficient to promote transdifferentiation when both are expressed outside of the pharynx and somatic gonad; PHA-4 regulates expression of at least one intestine-specific gene in collaboration with ELT-2 and has been shown to bind to the *elt-2* gene in vitro (Azzaria et al., 1996; Anokye-Danso et al., 2008). Further, FoxA is known to cooperate with GATA factors to promote gene expression in mammalian endodermal organs (reviewed in Zaret (1999)). We ectopically expressed *pha-4* under the control of a heat-shock promoter and confirmed the presence of immunoreactive PHA-4 outside of the pharynx and gonad (Fig. S2). Ectopic PHA-4 alone did not induce any clear developmental defects or ectopic intestine or pharynx formation at any stage of postembryonic development (Fig. S2). We next tested simultaneous expression of *pha*-*4* and *elt-7* under the same conditions and observed ectopic intestine markers only in the pharynx and somatic gonad, as was observed with ELT-7 expression alone (Fig. S2). Mammalian FoxA regulates chromatin compaction (Cirillo et al., 2002; Fakhouri et al., 2010) and, in *C. elegans*, PHA-4 binds to pharyngeal targets and induces chromosome de-compaction prior to activation of gene expression (Fakhouri et al., 2010). It is therefore conceivable that if PHA-4 could mediate decompaction of chromatin in non-pharyngeal cell types that it might be a temporally restricted process. We found however that multiple sequential heat shocks spaced by several hours did not reprogram cells other than the pharynx and somatic gonad (n=31). The finding that PHA-4 does not appear to be sufficient in combination with ELT-7 to promote reprogramming into intestine suggests that other cell types may express inhibitors of transdifferentiation, or alternatively that the pharynx and somatic gonad may express additional factors that make these tissues permissive for transdifferentiation.

**Fig. 7:**
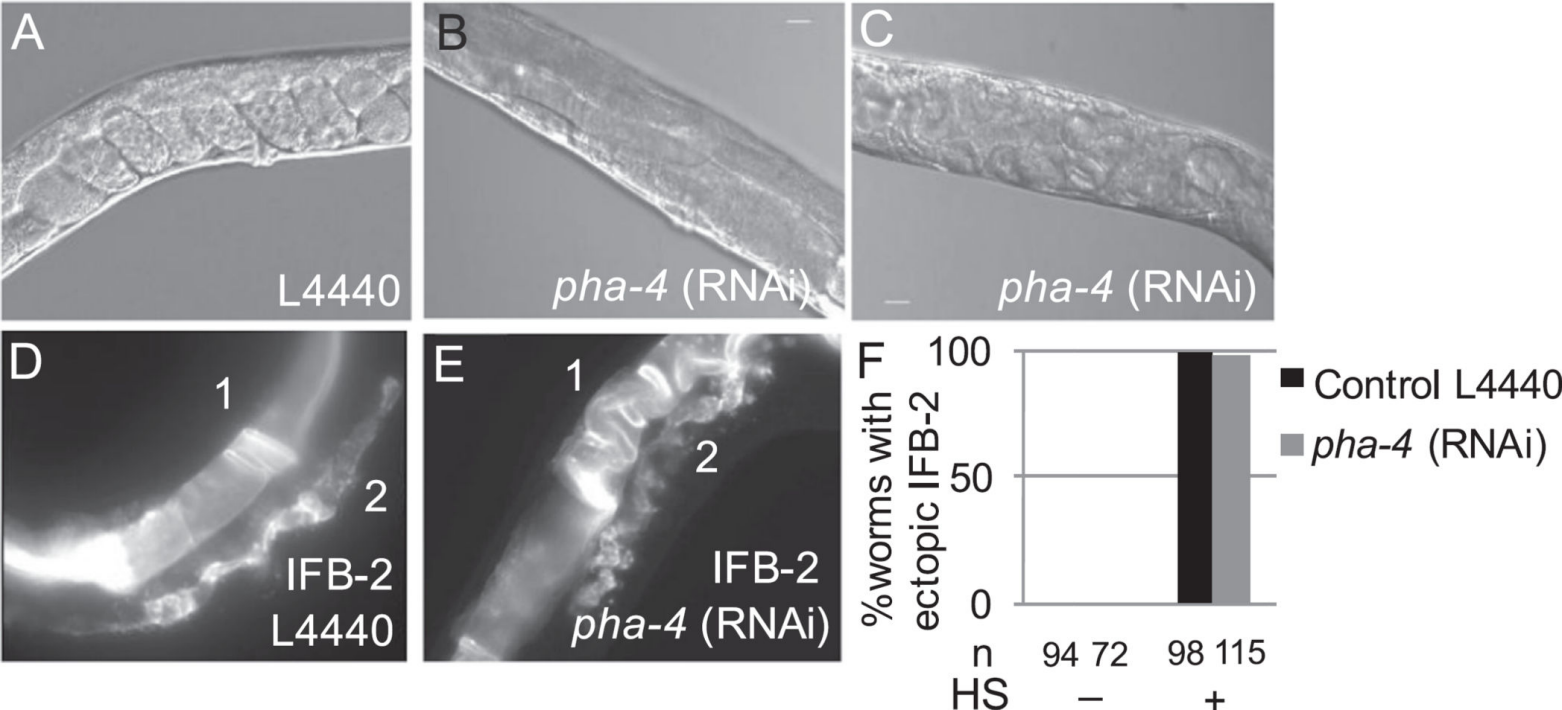
Post-embryonic PHA-4 is not necessary for transdifferentiation. (A) Proximal gonad of gravid adult from control (empty vector, L4440) RNAi feeding. (B–C) Proximal gonad of adult after *pha-4* RNAi feeding, (B) no embryos are visible, (C) many hatched larvae are present inside the worm, consistent with an egg laying defect. (D–E) IFB-2::CFP expression in the proximal gonad of worm from control (D) and *pha-4* RNAi (E) feeding conditions 48 h after pulsed ELT-7 expression at the L3 stage. IFB-2::CFP is visible in the intestine “1” and proximal gonad “2” under both conditions. (F) Percentage of worms with ectopic IFB-2::CFP in both the pharynx and somatic gonad under the indicated experimental conditions. HS, heat shock to induce ELT-7. n, number of worms viewed.

We found that normal post-embryonic PHA-4 function is also not required for transdifferentiation in the pharynx or for transorganogenesis of the uterus. We knocked down *pha-4* in larvae by feeding-mediated RNAi, which diminished *pha-4* reporter expression (not shown) and disrupted formation of the egg-laying apparatus in all larvae (Fig. 7, n=72). Nearly half of the worms failed to produce embryos (Fig. 7B) and others produced embryos that hatched within the adult (Fig. 7C). Ectopic ELT-7 expression following post-embryonic PHA-4 knock down did not prevent intestine-specific gene expression and redirection of pharynx and uterus development (Fig. 7D–F, n=115). Our findings suggest that post-embryonic PHA-4 is not necessary for reprogramming induced by ELT-7, with the caveat that low levels of PHA-4, not eliminated by RNAi, may be sufficient for the effect.

**Fig. 8:**
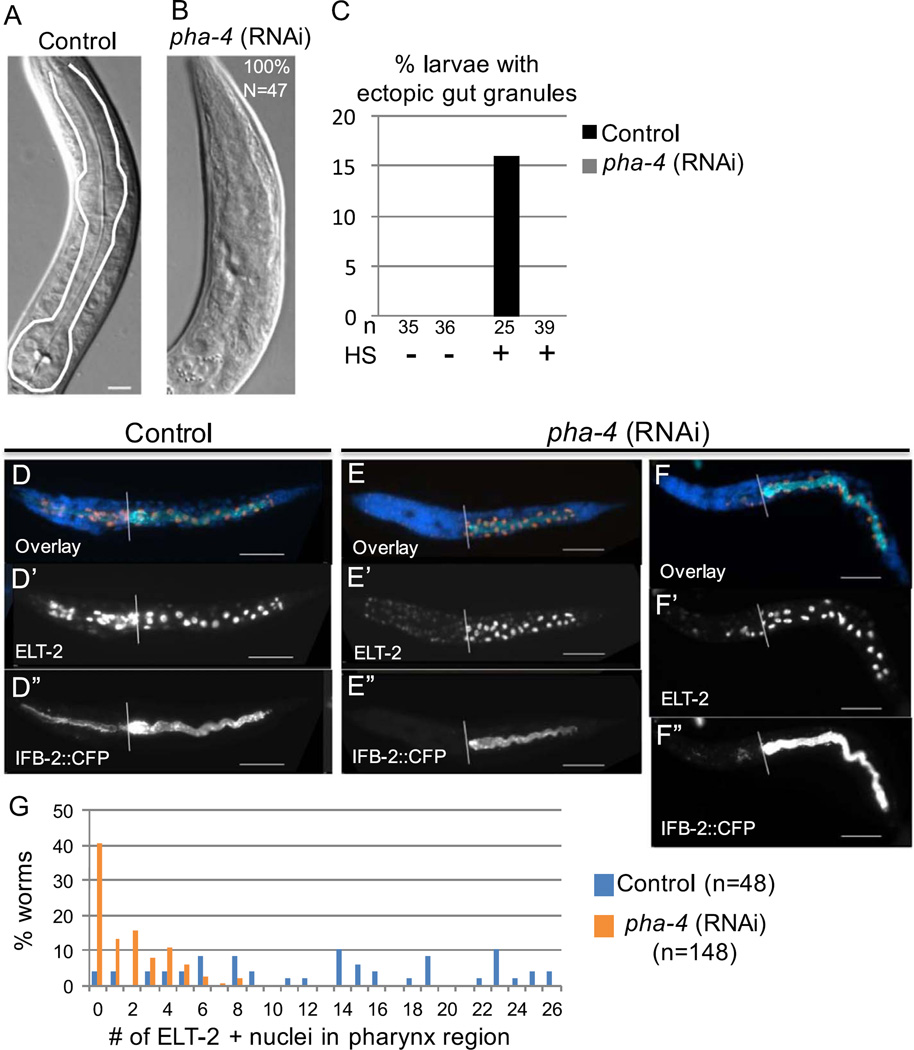
Pharynx differentiation is required for transdifferentiation into intestine. (A) The pharynx (outlined in white) in a hatched worm from control (empty vector, L4440) RNAi conditions. (B) Pharynx region after *pha-4*-targeted-embryonic RNAi; all worms completely lack a pharynx structure (n=47). (C) Percentage of worms from control or *pha-4* RNAi conditions with intestine-specific “gut granules” anterior to the normal intestine several days after ectopic ELT-7 expression. HS, heat shock. n, number of worms. (D–F) ELT-2 and IFB-2 expression in hatched worms 24 h after ectopic ELT-7 expression. Anterior is to the left and the white line marks the beginning of the intestine. Most worms lack ELT-2 or IFB-2 in the pharynx region after *pha-4* RNAi (E–E’); however, a fraction of worms contain a small number of ELT-2- and IFB-2-positive cells in the pharynx region (F–F’). (G) Percentage of worms with ELT-2-positive nuclei in the region anterior to the normal intestine after control and *pha-4* RNAi and ectopic ELT-7 expression. n, number of worms with immunoreactive nuclei.

While PHA-4 appears to be neither necessary nor sufficient post-embryonically for transdifferentiation of pharynx and uterus, we found that embryonic PHA-4 is required for transdifferentiation of the pharyngeal cell lineage (Fig. 8). Null mutations in *pha-4*, or depletion of *pha-4* embryonic transcripts, abolish formation of the pharynx (Fig. 8B); while the pharynx precursor cells are born, they do not form an organized structure or express markers of differentiated pharynx. They instead express an ectodermal marker and show no other distinguishing morphological features (Horner et al., 1998). We hypothesized that these cells, which have not properly differentiated, may be subject to reprogramming. However, we found that L1 stage worms completely lacking a pharynx did not develop ectopic intestine in the region of the pharyngeal precursors after ELT-7 expression (Fig. 8C). In RNAi feeding control conditions, we observed persistent *elt-2* expression, ectopic gut granules, and remodeling of pharynx cells in response to ELT-7 expression (Fig. 8C, D, G). In contrast, we never observed ectopic gut granules following widespread ELT-7 expression in PHA-4-depleted worms (n=39), and only rarely observed faint ELT-2 and IFB-2 in the pharynx region (Fig. 8E – G). Although all PHA-4-depleted worms lacked any sign of a pharynx by Nomarski microscopy, we observed sporadic immunoreactive PHA-4 in a small number of cells in some worms. Worms with detectable PHA-4 (Fig. S3, n=71) had an average of 11 positive cells after *pha-4* RNAi, compared to 33 PHA-4-positive cells in control RNAi conditions (n=44; p <0.001). The minor remnant of PHA-4 expression may explain the infrequent appearance of intestinal markers in the pharynx region. Our findings suggest that cells from the pharyngeal lineage that do not express pha-4 are not competent to undergo transdifferentiation into intestine. Yuzyuk et al. (2009), similarly seeking to understand how cell context influences developmental plasticity, expressed the END-1 endoderm-promoting factor in *pha-4*-mutant embryos at late embryonic stages and observed no evidence of ectopic intestine differentiation. They concluded that the pharyngeal precursors cells that lack PHA-4 deploy mechanisms to inhibit cell reprogramming that are distinct from the programs that induce differentiation. In contrast, our findings indicate that PHA-4 expression and pharynx differentiation are required for reprogramming into intestine, albeit by a different endoderm-promoting transcription factor, ELT-7.

## 3. Conclusions

We found that cells of two organs, the pharynx and somatic gonad, of *C. elegans* can be reprogrammed and their development redirected into intestine-like organs by multiple endoderm-promoting GATA transcription factors. Cells of these organs are not broadly developmentally plastic, but are specifically competent for reprogramming into intestine. It is noteworthy that reprogramming is successful even after cells have become post-mitotic in the somatic gonad at the mid-late L4 stage. Thus, our observations reflect not only an example of in vivo transdifferentiation, but the redirection in the development of an entire organ (specifically the uterus and spermatheca) into another (the intestine), in a process we have called “transorganogenesis.” This conversion of the proximal somatic gonad into gut can be compared to other instances in which ectopic expression of a single gene induces ectopic organ formation. For example, in *Drosophila* expression of *eyeless* (Halder et al., 1995) and *dachshund* (Shen and Mardon, 1997) were shown to be sufficient to induce ectopic eyes on the antennae and thorax. The distinguishing feature of our findings in *C. elegans* is the stage at which the formation of the organ can be redirected. In the above-mentioned studies, forced expression of a central regulator leads to transdifferentiation from a relatively plastic set of immature imaginal disc cells, whereas we found that a single differentiation factor can redirect development even during the final, postmitotic stages of organogenesis. The redirected cells likely redeploy an embryonic gene regulatory network as the activation and timing of events parallel that of the normal embryonic program for gut development.

The intestine, somatic gonad, and pharynx are distantly related in lineage but they all form epithelial tubes with cells that express the PHA-4/FoxA transcription factor. We found that pharynx differentiation, orchestrated by PHA-4/FoxA, is required for transdifferentiation into intestine. FoxA transcription factors are expressed in tube-forming cells across metazoan phylogeny and may regulate an ancestral gene regulatory network that drives the developmental formation of tubular organs (reviewed in de-Leon (2011)). Differentiation of digestive tract modules is controlled by the collaboration of GATA and FoxA factors in animals spanning metazoan phylogeny from *C. elegans* to humans (Zaret, 1999; Cirillo et al., 2002; Anokye-Danso et al., 2008). Our finding that *C. elegans* foregut and somatic gonad cells can be converted to midgut cells by GATA factor expression implies that modules of the digestive or reproductive tract in other animals may be interconverted by post-embryonically modulating GATA transcription factor expression. It is interesting to note that metaplasias (the conversion of one tissue type into another) occur most frequently in the digestive and reproductive tracts of humans (Slack, 1985), and can involve transformations of the type we have observed. Barrett’s metaplasia, for example, is the conversion of squamous epithelium of the esophagus into cuboidal intestine-like epithelium and is associated with changes in gene expression that can lead to esophageal cancer (Slack et al., 2010). Understanding how cell context influences susceptibility to changes in cell identity in the digestive and reproductive tracts could lead to treatments for cancers or methods for producing patient-specific cells. Our findings highlight the importance of cell context in determining susceptibility to developmental reprogramming and have established an in vivo model to investigate the mechanisms that influence the redirection of organ identity.

## 4. Methods

### 4.1. Nematode strains, maintenance, and heat shock

Nematode strains were maintained as described (Sulston and Hodgkin, 1988) and experiments were carried out at 20 °C unless noted. The following strains were used: JR3410 *wIs47[hsp-16-2::end-1, hsp-16–41::end-3; rrIs01[elt-2::lacZ::GFP; unc-119(+)]* (Kostic and Roy, 2002; Fukushige et al., 1998; Zhu et al., 1998), JR3402 *wIs76[hsp-16-2::end-3, hsp-16–41::end-3]; rrIs01* (Djabrayan et al., 2012), JR3373 *wIs125[hsp-16-2::elt-7 hsp-16–41::elt-7]; rrIs01* (Sommermann et al., 2010), JR3405 *caIs8[hsp16-2::elt-2, hsp16-41::elt-2]; rrIs01* (Gilleard and McGhee, 2001), JR3646 *wIs125[hsp-16-2::elt-7 hsp-16–41::elt-7]; him-5(e1490); kcIs6[IFB-2::CFP]* (*kcIs6* kindly provided by Olaf Bossinger) (Husken et al., 2008), JR3339 [ccIs4251 (*myo-3::GFP*), *hs-hlh-1* (KM438, Fukushige and Krause, 2012]. JG7 *caIs6* [*hsp16-2::elt-1* + pRF4 [*rol-6(su1006dm*)] ; *ijIs12[dpy-7::GFP*] (Gilleard and McGhee, 2001), JR3649 *wIs125; kcIs6; caIs18[pha-4::GFP]*, JR3691 *kcIs6[ifb-2::CFP]; cgc539Is1[hsp16.2::pha-4]*, JR3642 *wIs125[hsp-16-2::elt-7 hsp-16–41::elt-7]; rrIs01[elt-2::lacZ::GFP; unc-119(+)]; icIs6[ifb-2::cfp]*. To express ELT-7 and PHA-4 simultaneously we crossed males of JR482 (*wIs125; him-5(e1490)*; *kcIs6[IFB-2::CFP])* to hermaphrodites of JM70 (*cgc5395Is1[hsp16.2::pha-4]* (Kalb et al., 1998b)), and heat shocked progeny that expressed IFB-2::CFP.

To examine the timeline of ectopic intestine marker expression, we heat-shocked L3 and mid L4 stage worms on agar plates at 33 °C for 30 min. For each of 50 worms, the presence of ectopic GFP and CFP in the pharynx, gonad, or in “other” tissues was scored. Time points are +/− 30 min owing to time required for scoring. We determined the developmental stages susceptible to somatic gonad transorganogenesis by mounting single worms onto agar pads, recording their length and stage of vulva development, immediately heat shocking the worms for 15 min on agar pads in a 33 °C incubator, and moving the worms to individual NGM plates seeded with OP50. Worms were viewed after 24–48 h using a Nikon Eclipse Ti inverted microscope. Images were taken with a Hamamatsu flash Orca 2.8 camera. Brightness and contrast of some images have been adjusted to better show relevant details in print versions.

### 4.2. Transmission electron microscopy

Worms anesthetized with 8% ethanol were examined for formation of an ectopic intestine-like lumen structure. Worms displaying an advanced phenotype were transferred to buffered aldehyde (2.5% glutaraldehyde, 1% formaldehyde, 0.2 M sucrose, 1 mM MgCl_2_, 0.05 M cacodylate) and immediately decapitated, then incubated overnight at 4 °C. Fixed worm pieces were washed five times in cacodylate buffer, stained with 1% buffered osmium tetroxide for one hour at room temperature, washed five times in cacodylate buffer, then embedded in 2.5% agarose. Agarose slabs were dehydrated with five-minute washes in 30%, 50%, 70%, then 100% ethanol. Samples were then washed several times in 100% propylene oxide, and progressively infiltrated into Embed812 resin (Hall et al., 2012). After infiltration into plastic resin, samples were flat embedded between Aclar sheets, then cured at 60°C for two days. Single worms were viewed under the dissecting microscope, cut out of the Aclar sandwich before re-embedding in fresh plastic resin and placed in a mold in precise orientation followed by curing again at 60°C. The embedded sample was trimmed with a razor blade and serial thin-sectioned on an RMC PowerTome XL, using a diamond knife. Sections were mounted on Pioloform-coated slot grids, post-stained with uranyl acetate, and viewed with a Philips CM10 electron microscope. Digital images were collected using an SIS camera system and viewed using iTEM or Photoshop software platforms to analyze data and select images for illustrations.

### 4.3. Immunohistochemistry

Larvae were synchronized as described (Stiernagle, 2006) and heat shocked at the desired stage in M9 buffer at 33 °C for 15 min using a thermal cycler. Anti-ELT-2 antibody was a gift of J. McGhee (University of Calgary, Canada). Anti-PHA-4 antibody was a gift of S. Mango (Harvard, MA). Cy3 goat anti-mouse and Cy3 goat anti-rabbit was obtained from Sigma. Fixation and permeabilization of L2-adult (Finney and Ruvkun, 1990) or L1 (Sommermann et al., 2010) stage worms was carried out as described.

### 4.4. RNAi feeding

Control L4440 (empty vector) or *pha-4* RNAi bacterial strains (Kamath et al., 2003) were grown overnight at 37 °C in 3 mL LB containing ampicillin (100 µg/mL). 200 µL of the overnight culture was added to 2 mL LB containing ampicillin (100 µg/mL). After 4 h of incubation at 37 °C, IPTG was added to the culture to a final concentration of 1 mM and 100 µL was seeded onto 35 mm agar plates containing 1 mM IPTG. Seeded plates were allowed to dry at room temperature then incubated for 24 h at 37 °C. To investigate embryonic PHA-4 function (embryonic-targeted RNAi), L4 stage worms were transferred to RNAi feeding, allowed to feed for 24–48 h and the embryos were isolated using bleach (Stiernagle, 2006). Embryos were hatched in M9 buffer at 15 °C and heat shocked at 33 °C using a thermal cycler. We examined post-embryonic RNAi phenotypes by feeding synchronized L1 stage worms for 2–3 days and heat shocking at L3 to mid-L4 on 35 mm NGM plates for 30 min at 33 °C.

## Supplementary Material

1

2
