# Supplementary material for: Transorganogenesis and transdifferentiation in C. elegans are dependent on differentiated cell identity
Source: Dev Biol. Author manuscript; Available in PMC 2017 Jan 10. (PMC5224929; doi:10.1016/j.ydbio.2016.09.020)

Supplemental figure 1

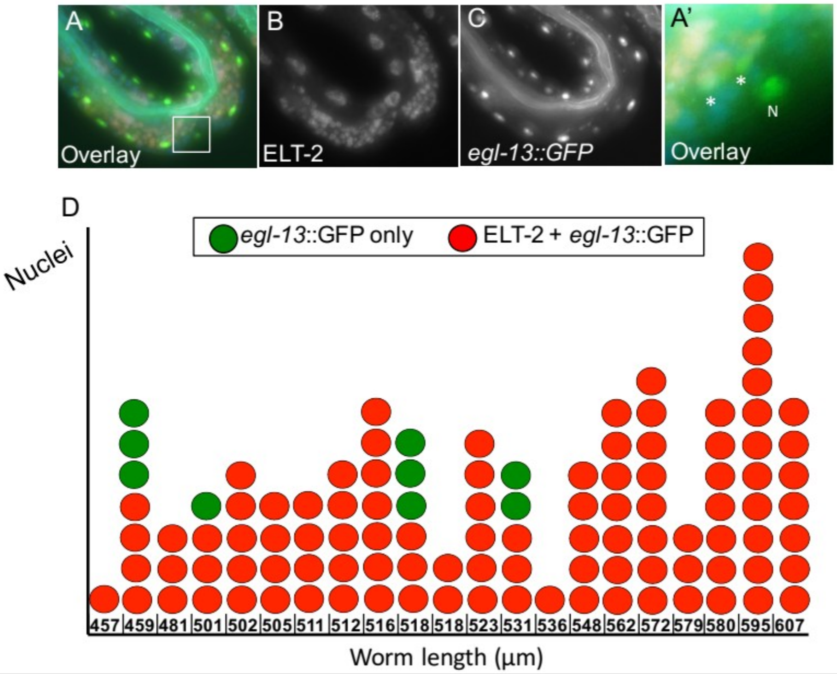

Supplemental figure 2

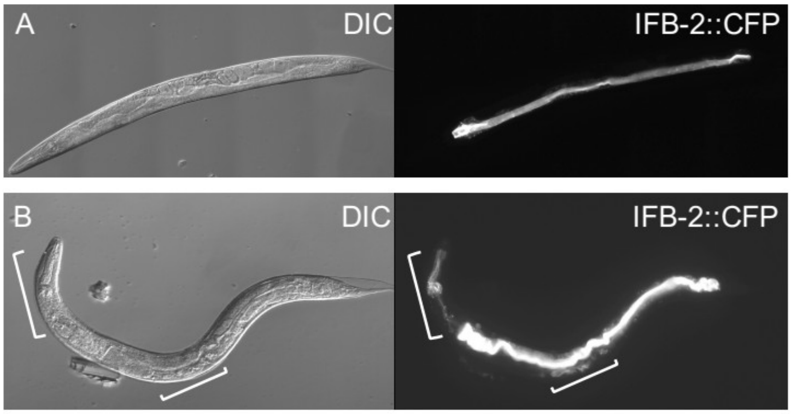

Supplemental Figure 3

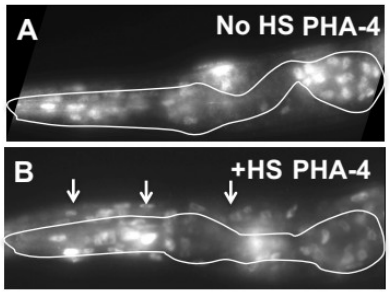

Supplemental Figure 4

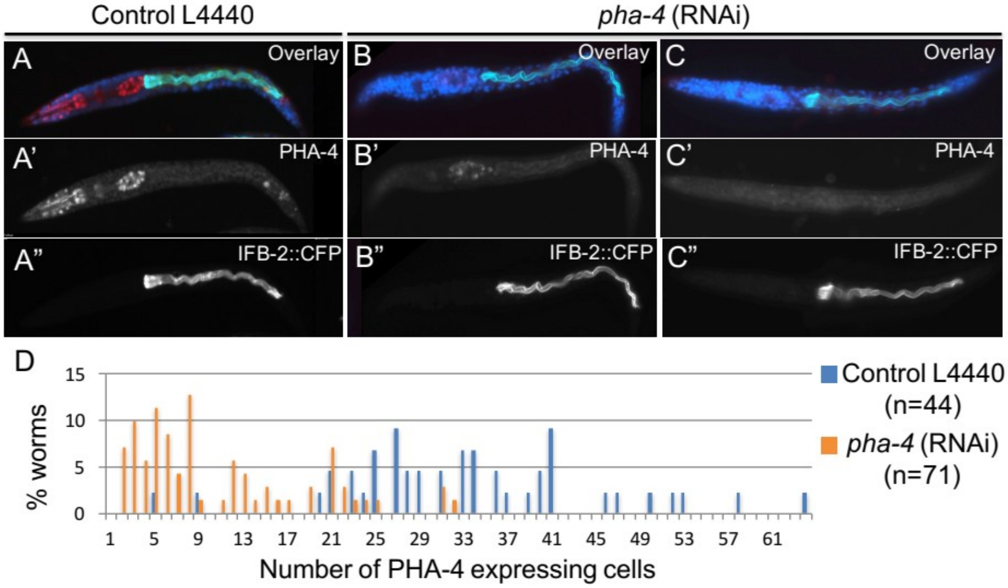

Supplement: Supplementary file 1 [file NIHMS837010-supplement-1.docx]
